# Acid-Sensing Ion Channel 1a Is Involved in N-Methyl D-Aspartate Receptor-Dependent Long-Term Depression in the Hippocampus

**DOI:** 10.3389/fphar.2019.00555

**Published:** 2019-05-21

**Authors:** D. Mango, R. Nisticò

**Affiliations:** ^1^ Laboratory of Neuropharmacology, European Brain Research Institute, Rita Levi-Montalcini Foundation, Rome, Italy; ^2^ Department of Biology, School of Pharmacy, University of Rome Tor Vergata, Rome, Italy

**Keywords:** ASIC, hippocampus, electrophysiology, LTD, NMDA receptors

## Abstract

Acid-sensing ion channels (ASICs), members of the degenerin/epithelial Na^+^ channel superfamily, are largely expressed in the mammalian nervous system. ASIC1a is highly permeable to Ca^2+^ and are involved in many physiological processes, including synaptic plasticity, learning, and memory. To clarify the role of ASIC1a in synaptic transmission and plasticity, we investigated N-methyl D-aspartate (NMDA) receptor-dependent long-term depression (LTD) in the CA1 region of the hippocampus. We found that: (1) ASIC1a mediates a component of ASIC1a excitatory postsynaptic currents (EPSCs); (2) ASIC1a plays a role in electrical LTD induced by LFS protocol both in P13-18 and P30-40 animals; (3) ASIC1a is involved in chemical LTD induced by brief bath application of NMDA both in P13-18 and P30-40 animals; and finally (4) a functional interaction between ASIC1a and NMDA receptors occurs during LTD. These findings suggest a new role for ASIC1a in specific forms of synaptic plasticity in the mouse hippocampus.

## Introduction

Acid-sensing ion channels (ASICs), members of the degenerin/epithelial Na^+^ channel superfamily, are largely expressed in the mammalian nervous system (Waldmann et al., 1997a). ASIC1a is highly permeable to Ca^2+^ and localized in different brain regions with high synaptic density, including the hippocampus (Baron et al., 2002; Askwith et al., 2004; Weng et al., 2010; Sherwood et al., 2011). ASIC1a is present at excitatory postsynaptic sites, being activated under normal and pathological conditions (Wemmie et al., 2003, 2006; Zha et al., 2006). In the last years, a growing body of evidence has shown that acidosis, through the activation of ASICs, contributes to synaptic plasticity, i.e., long-term potentiation and dendritic structural plasticity in hippocampal neurons, as well as learning and memory (Wemmie et al., 2002; Xiong et al., 2004; Zha et al., 2006; Arias et al., 2008; Vergo et al., 2011; Du et al., 2014; Kreple et al., 2014; Buta et al., 2015; Mango et al., 2017).

Extracellular acidification occurs in the brain during many different physiological and pathological situations as elevated neural activity, increased metabolism, and neuronal injury. Several works suggested that the acidic pH of synaptic vesicles transiently influences local extracellular pH during neurotrasmitters release (Krishtal et al., 1987; Waldmann et al., 1997b). According with this idea, it has been demonstrated that transient acidification of extracellular pH occurs in synaptic transmission in cultured hippocampal neurons (Miesenböck et al., 1998) and also in hippocampal slices (Krishtal et al., 1987; Wemmie et al., 2003, 2006). In light of these evidences, it has been proposed that ASICs provide a target for protons released in neurotransmission and thus play a role in the physiology of synaptic function (Krishtal et al., 1987; Waldmann et al., 1997b; Du et al., 2014).

Several observations suggest that ASICs contribute to synaptic plasticity in different brain areas. Specifically ASICs facilitate activation of the N-methyl D-aspartate (NMDA) receptor during LTP induction, suggesting a functional interaction between these receptors in the regulation of hippocampal synaptic plasticity (Wemmie et al., 2002; Du et al., 2014; Buta et al., 2015; Liu et al., 2016). Also, the coupling between NMDA receptor and ASIC has been shown to exacerbate acidity-mediated neuronal death occurring during ischemia (Gao et al., 2005, 2015). More recently, our group has suggested a novel function of ASIC1a in the modulation of group I mGlu receptor-dependent synaptic plasticity and intrinsic excitability in the hippocampus (Mango et al., 2017). To shed some light on the role of ASIC1a in another form of synaptic plasticity, here we performed an electrophysiological analysis to explore ASIC1a involvement in excitatory synaptic transmission and in NMDA receptor-dependent LTD in young and adult mice.

## Materials and Methods

This study was carried out in accordance with the recommendations of international guidelines on the ethical use of animals from the European Communities Council Directive (2010/64/EU). The protocol was approved by the Ministero della Salute.

### Slice Preparation

C57BL6/J mice (13–40 days old) were deeply anesthetized with isoflurane and killed by decapitation.

The brain was rapidly removed from the skull and parasagittal hippocampal slices (250 μm) containing the dorsal hippocampus were cut with a vibratome (VT 1200S, Leica) in cold (0°C) artificial cerebrospinal fluid (aCSF) containing (in mM): NaCl (124); KCl (3); MgSO_4_ (1); CaCl_2_ (2); NaH_2_PO_4_ (1.25); NaHCO_3_ (26); glucose (10); saturated with 95% O_2_, 5% CO_2_ (pH 7.4), and left to recover for 1 h in ACSF at 33.5°C.

### Electrophysiology

Individual slices were placed in a recording chamber, on the stage of an upright microscope (Nikon, Japan) and submerged in a continuously flowing (3 ml/min) solution at 28°C (±0.2°C). Individual neurons were visualized through a 40× water-immersion objective (Nikon, Japan) connected to infrared video microscopy (Hamamatsu, Japan). Borosilicate glass electrodes (3–7 MΩ), pulled with a PP 83 Narishige puller, were filled with a solution containing the following (in mM): CsCl (135), KCl (10), CaCl_2_ (0.05), EGTA (0.1), Hepes (10), Na3-GTP (0.3), Mg-GTP (4.0), pH adjusted to 7.3 with CsOH or K-Gluconate (135); KCl (10); MgCl_2_ (2); CaCl_2_ (0.05); EGTA (0.1); HEPES (10); ATP (4); GTP (0.3), pH 7.3 with KOH as previously published (Mango et al., 2017).

Whole-cell voltage clamp (at −70 mV holding potential) recordings were carried out with a MultiClamp 700B amplifier (Axon Instruments, Foster City, CA), filtered at 1 kHz and digitized (10 kHz).

The excitatory postsynaptic currents (EPSCs) were elicited at 0.033 Hz (EPSCs were elicited by stimulation every 30 s and each plot represents the mean of two consecutive EPSCs) with a glass pipette filled with ACSF, placed in CA1 *stratum radiatum* to stimulate Shaffer collateral fibers and in continuous presence of picrotoxin in bath solution as previously published (Mango et al., 2016). For paired-pulse (PP) experiments, paired-pulse stimuli (50 ms inter-pulse interval) were elicited with stimulating electrode placed close to the recording neuron in the continuous presence of picrotoxin. Paired pulse ratio (PPR) was evaluated as ratio of second EPSC amplitude on first EPSC amplitude.

Low-frequency stimulation (LFS) was obtained by delivering 300 pulses (at 0.75 Hz) at −40 mV.

The ASIC1a inward current was elicited by a CSF buffered to pH 5.5 and pressure-applied (10 psi, 1 s) through a patch electrode connected to Pneumatic Pico-Injector. The puff electrode was positioned above the slice in close proximity to the recorded neuron, and the inward currents were elicited every 3 min.

Electrophysiological data are represented as mean values ± SEM. Statistical significance was evaluated by two-tailed Student’s *t*-test on averaged mean values taken from the last 5 min of each experiment. Statistical significance was set at *p* < 0.05 (indicated in the figures by *).

### Drugs

Amiloride; 6-cyano-7-nitroquinoxaline-2,3-dione (CNQX); D-2-amino-5-phosphonovaleric acid (D-AP5); isoflurane; picrotoxin; and N-methyl-d-aspartic acid (NMDA) were purchased from Sigma-Aldrich (Italy); and psalmotoxin-1 was purchased from Alomone Labs. All drugs were dissolved in water.

## Results

### Acid-Sensing Ion Channel 1a-Mediated Currents in CA1 Pyramidal Neurons

**Figure 1:**
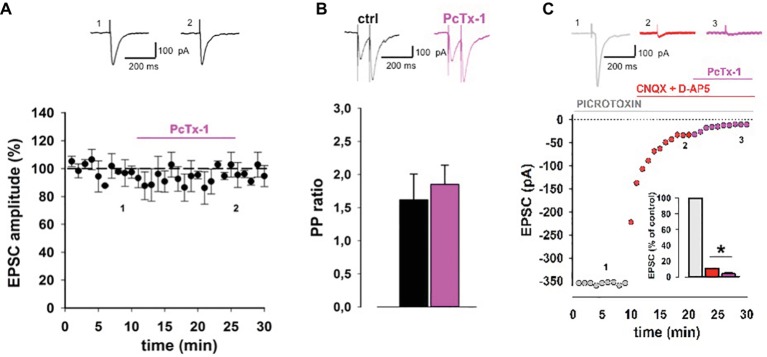
ASIC1a contributes to excitatory synaptic transmission in hippocampal slices. **(A)** Normalized pooled data showing EPSCs in control condition and during PcTx-1 (100 ng/ml) bath application (mean ± SEM, *n* = 12, *p* > 0.05). EPSC was elicited by monopolar electrode placed on Schaffer collateral fibers. On top, representative EPSC traces were taken at the time indicated by number. **(B)** Bar chart illustrates normalized PPR (50 ms inter-pulse interval) in control condition and during PcTx-1 (100 ng/ml) bath application (mean ± SEM, *n* = 8, *p* > 0.05). On top, representative EPSC traces are shown. **(C)** Slices were perfused with picrotoxin (GABA_A_ receptor antagonist, grey), plus CNQX (30 μM; AMPA receptor antagonist) and D-AP5 (50 μM; NMDA receptors antagonist, red), and subsequently with 100 ng/ml PcTx-1 (pink). Panel shows one representative experiment, bar chart in the inset illustrates the amplitude of EPSC (% of control) in the different conditions (mean ± SEM, *n* = 6, *p < 0.05).

In order to assess whether ASIC1 contributes to basal excitatory transmission, we recorded excitatory postsynaptic currents (EPSCs) elicited by stimulation of the Schaffer collaterals fibers in the presence of PcTx-1, a polypeptide from the venom of spider toxin, that specifically inhibits homomeric ASIC1a currents (Escoubas et al., 2000). Bath application of PcTx-1 (100 ng/ml) did not induce any significant change in EPSC amplitude (*p* > 0.05, *n* = 12; Figure 1A). Also PPR, which is used to investigate the probability of neurotransmitter release, was unaffected following bath application of PcTx-1 (*p* > 0.05, *n* = 8; Figure 1B), according to a postsynaptic localization of ASIC1a (Fioravante and Regehr, 2011). While protons activate an inward current in different brain areas (Du et al., 2014; Highstein et al., 2014; Kreple et al., 2014), we then investigated the impact of proton-activated current on the EPSC in CA1 pyramidal neurons. Application of the ionotropic glutamate receptor antagonists CNQX and D-AP5 strongly reduced EPSC amplitude to 10.6 ± 3% of control (*p* < 0.05, *n* = 6; Figure 1C). In this condition, application of PcTx-1 (100 ng/ml) further reduced the current to 4 ± 2% of control (*p* < 0.05, *n* = 6; Figure 1C). The amplitude of the ASIC1a-mediated current is very small and this explains why the effect of PcTx-1 is masked in baseline excitatory neurotransmission.

### Acid-Sensing Ion Channel 1a Contributes to Hippocampal N-Methyl D-Aspartate Receptor-Dependent Long-Term Depression in Young Mice

We next investigated the involvement of ASIC1a in a specific form of NMDA receptor-dependent synaptic plasticity. It is known that low-frequency stimulation protocol induces a stable long-term depression of synaptic transmission (Dudek and Bear, 1993; Nicolas et al., 2012). The induction of this form of LTD was completely inhibited by the NMDA receptor antagonist D-AP5 (50 μM) (data not shown).

**Figure 2:**
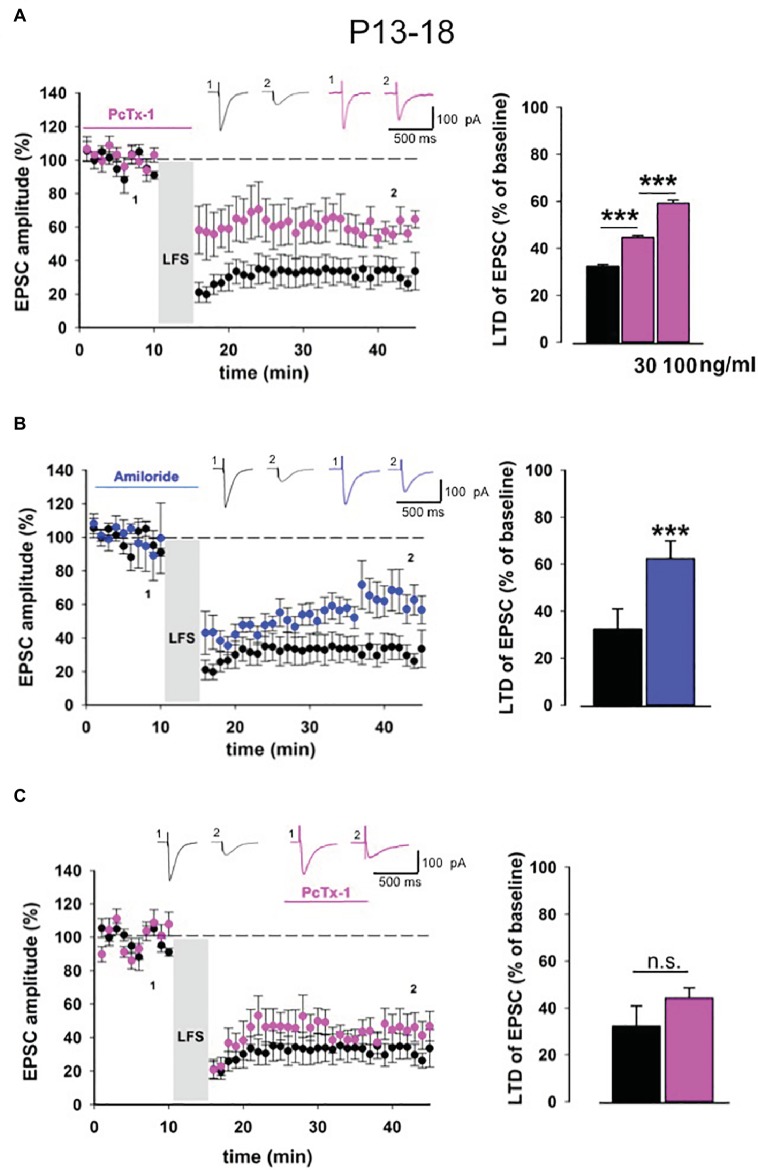
ASIC1a modulates NMDA receptor-dependent LTD in young mice. **(A)** Normalized pooled data showing LTD in control condition (black) and in the presence of Pctx-1 applied before and during LFS (100 ng/ml, pink). Histogram represents the last 5 min of experiment in control (*n* = 12) condition or in the presence of PcTx-1 (30 ng/ml, mean ± SEM, *n* = 10, ***p < 0.001), (100 ng/ml, mean ± SEM, *n* = 10, ***p < 0.001). On top, representative traces are shown for each condition. **(B)** Normalized pooled data showing LTD in control condition (black) and in the presence of amiloride (100 μM, blue). Histogram represents the last 5 min of experiment in control condition (*n* = 12) or in presence of amiloride (*n* = 8) (mean ± SEM, ***p < 0.001). **(C)** Normalized pooled data showing LTD in control condition (black) and in the presence of PcTx-1 (100 ng/ml, pink) applied after LFS protocol. Histogram represents the last 5 min of experiment in control condition (*n* = 12) or in the presence of PcTx-1 (*n* = 6) (mean ± SEM, *p* > 0.05).

We performed LTD experiments in young mice (P13-18) in the presence of PcTx-1 at different concentrations, applied 10 min before and during LFS protocol. PcTx-1 was able to reduce, in a dose-dependent manner, the magnitude of EPSC-LTD compared to control conditions (control: 32.2 ± 0.9% of baseline, *n* = 12; PcTx-1 30 ng/ml: 44.5 ± 0.9% of baseline, *n* = 10, *p* < 0.001; PcTx-1,100 ng/ml: 59.1 ± 1.3% of baseline, *n* = 10, *p* < 0.001, Figure 2A).

To further confirm that ASIC1a is involved in this form of synaptic plasticity, we also performed experiments using the nonselective ASIC blocker amiloride (100 μM). Even under this condition, LTD magnitude was significantly reduced compared to control condition (62.3 ± 1.8% of baseline, *n* = 8, *p* < 0.001, Figure 2B).

To distinguish whether ASIC1a plays a role in the induction or rather in the maintenance phase of LTD, we bath applied PcTx-1 10 min after the induction of LTD. Notably, in this condition PcTx-1 did not affect LTD magnitude (44.2 ± 0.8% of baseline, *n* = 6, *p* > 0.05, Figure 2C).

### Acid-Sensing Ion Channel 1a Contributes to Hippocampal N-Methyl D-Aspartate Receptor-Dependent Long-Term Depression in Adult Mice

**Figure 3:**
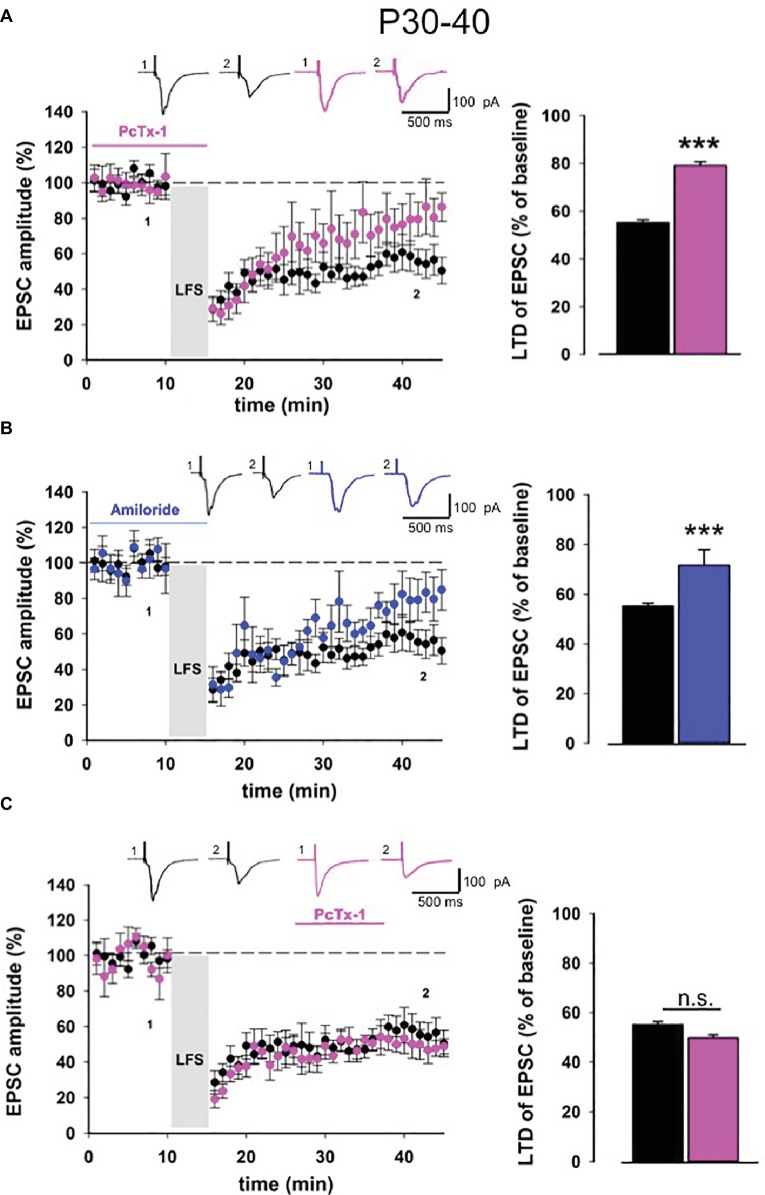
ASIC1a modulates NMDA receptor-dependent LTD in adult mice. **(A)** Normalized pooled data showing LTD in control condition (black) and in the presence of Pctx-1 before and during LFS (100 ng/ml, pink). Histogram represents the last 5 min of experiment in control condition (*n* = 11) or in the presence of PcTx-1 (*n* = 9) (mean ± SEM, ***p < 0.001). On top, representative traces are shown for each condition. **(B)** Normalized pooled data showing LTD in control condition (black) and in the presence of amiloride (100 μM, blue). Histogram represents the last 5 min of experiment in control condition (*n* = 11) or in the presence of amiloride (*n* = 7) (mean ± SEM, ***p < 0.001). **(C)** Normalized pooled data showing LTD in control condition (black) and in the presence of PcTx-1 (100 ng/ml, pink) after LFS. Histogram represents the last 5 min of experiment in control condition (*n* = 11) or in the presence of PcTx-1 (*n* = 8) (mean ± SEM, *p* > 0.05).

Long-term depression is strongly affected by the developmental stages of the brain (Kemp and Bashir, 1997; Kemp et al., 2000). Thus, we extended our electrophysiological analysis also in slices obtained from adult mice (P30-40). Similar to young mice, in adult mice also, LTD magnitude was reduced when PcTx-1 (100 ng/ml) was applied (control: 55.1 ± 1.2% of baseline, *n* = 11, *p* < 0.001, PcTx-1: 79.1 ± 1.5% of baseline, *n* = 9, *p* < 0.001, Figure 3A). A comparable effect was also obtained in the presence of amiloride (100 μM) (81 ± 1% of baseline, *n* = 7, *p* < 0.001 Figure 3B). Furthermore, we applied PcTx-1 after LTD induction and, as it occurred in young mice, no change was observed in LTD magnitude compared to control (49.7 ± 1.4% of baseline, *n* = 8, *p* > 0.05, Figure 3C).

### PcTx-1 Inhibits Chemical N-Methyl D-Aspartate Receptor-Dependent Long-Term Depression

**Figure 4:**
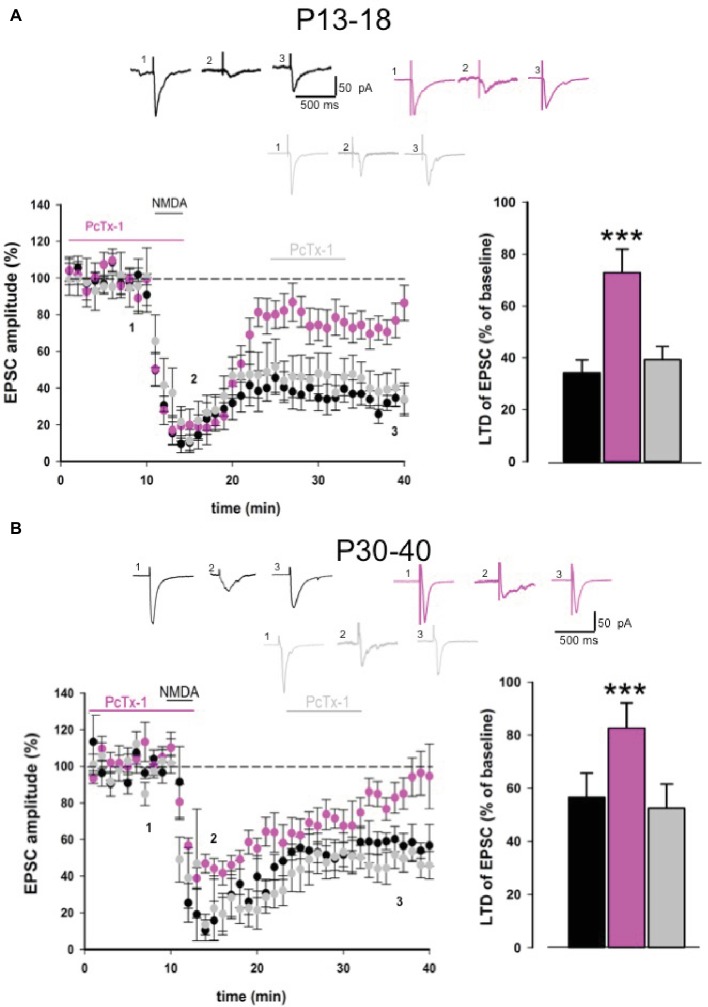
PcTx-1 inhibits chemical NMDA-LTD. **(A)** Pooled data showing LTD induced by NMDA (20 μM) bath applied in slices obtained from young mice in control condition (*n* = 9) (black), in the presence of PcTx-1 (*n* = 7) (100 ng/ml, pink) applied before and during NMDA application, or applied after LTD induction (*n* = 6) (grey). Histogram represents the last 5 min of experiment in each condition (mean ± SEM, ***p < 0.001). On the top, representative traces are shown for each condition. **(B)** Pooled data showing LTD induced by NMDA (20 μM) bath applied in slices obtained from adult mice in control condition (*n* = 8) (black), in the presence of PcTx-1 (*n* = 7) (100 ng/ml, pink) applied before and during NMDA application, or applied after LTD induction (*n* = 5) (grey). Histogram represents the last 5 min of experiment in each condition (mean ± SEM, ***p < 0.001). On the top, representative traces are shown for each condition.

To further confirm the role of ASIC1a in NMDA receptor-dependent LTD, we performed experiments using a chemical protocol based on the perfusion of NMDA on hippocampal slices obtained from young and adult mice (Kamal et al., 1999). Bath application of NMDA (20 μM, 3 min) on hippocampal slices from young animals induced a strong EPSC depression that was detectable 30 min after NMDA application (34.1 ± 5.1% of baseline, *n* = 9, Figure 4A). Application of PcTx-1 (100 ng/ml) 10 min before and during the perfusion of NMDA partially rescued LTD (72.9 ± 9% of baseline, *n* = 7, *p* < 0.001, Figure 4A), suggesting that ASIC1a plays a modulatory role also in this form of LTD. Notably, bath application of PcTx-1 after NMDA-LTD induction did not restore the established depression of EPSC (39.4 ± 5% of baseline, *n* = 6, *p* > 0.05, Figure 4A).

Similar results were obtained also in slices from adult mice. Indeed, NMDA perfusion induced a strong EPSC depression (56.6 ± 9% of baseline, *n* = 8, Figure 4B). Pharmacological blockade of ASIC1a before and during NMDA application rescued LTD (82.7 ± 9.5% of baseline, *n* = 7, Figure 4B). On the contrary, when PcTx-1 was applied after NMDA-LTD induction, LTD still persisted (52.4 ± 9.2% of baseline, *n* = 5, Figure 4B), suggesting that ASIC1a modulates NMDA receptor function *per se*, thereby affecting NMDA receptor-mediated LTD maintenance signaling.

### PcTx-1 Converts Long-Term Depression of EPSC_NMDA_ to Potentiation

To explore the functional interplay between ASIC1a and NMDA receptors, we performed LTD experiments on the EPSC_NMDA_, pharmacologically isolated through bath application of picrotoxin and CNQX. Although here we refer to an EPSC_NMDA_ component, we cannot exclude, based on our previous results, that also ASIC1a may be contributing to this current.

**Figure 5:**
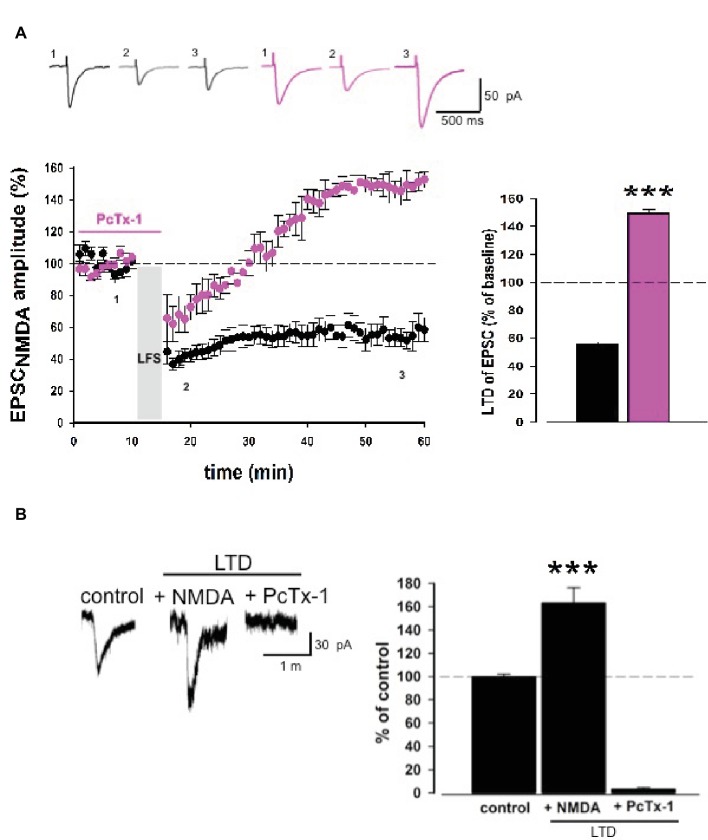
PcTx-1 converts LTD of EPSC_NMDA_ to LTP. **(A)** Normalized pooled data showing LTD of EPSC_NMDA_ in control condition (black) and in the presence of PcTx-1 before and during LFS (100 ng/ml, pink). Histogram represents the last 5 min of experiment in control condition (*n* = 9) or in the presence of PcTx-1 (*n* = 7) (mean ± SEM, ***p < 0.001). On top, representative traces are shown for each condition. **(B)** Histogram represents the amplitude of ASIC1a-mediated inward current (control condition), in the presence of NMDA up to 10 min later, and in the presence of PcTx-1 (*n* = 6, mean ± SEM, ***p < 0.001). On the left, representative traces are shown for each condition.

Low-frequency stimulation produces a stable long-term depression of EPSC_NMDA_. Surprisingly, PcTx-1 applied before and during LFS was able to convert LTD to LTP (control: 55.4 ± 2.5% of baseline, *n* = 9; PcTx-1,100 ng/ml: 149.3 ± 3.2% of baseline, *n* = 7, *p* < 0.001, Figure 5A), even though the mechanisms underlying this effect need to be explored.

To investigate possible changes of ASIC-mediated currents following LTD induction, we performed experiments by delivering puff applications of ACSF at pH 5.5 which elicit inward currents mediated by ASIC1a. Puff applications were applied at a 3-min interval for 10 min before and 10 min after NMDA-LTD induction (NMDA 20 μM, 3 min). Interestingly, we observed an increase in the amplitude of ASIC inward current following NMDA-LTD induction (163 ± 13.2%, *n* = 6, *p* < 0.001, Figure 5B), which was then completely abolished by PcTx-1 applied (control: 99.4 ± 2.5% vs. PcTx-1 2.8 ± 1.8%, *n* = 6 *p* < 0.001; Figure 5B). Overall, these data confirm that a functional interplay between ASIC1a and NMDA receptors underlies specific forms of synaptic plasticity.

## Discussion

In the present study, we have demonstrated that ASIC1a contributes, although to a small extent, to basal excitatory postsynaptic currents in CA1 pyramidal neurons. Indeed in hippocampal slices, the proton-mediated current had a little effect on neurotransmission under basal condition comparable to what was already shown in the amygdala (Du et al., 2014). As previously demonstrated, neurotransmitters glutamate and H^+^ are released together during excitatory synaptic transmission bringing a rapid pH drop at synaptic cleft which activates ASICs (Waldmann et al., 1997a,b; DeVries, 2001; Traynelis and Chesler, 2001; Xiong et al., 2004).

Some studies have investigated the involvement of ASIC1a in synaptic plasticity and have shown that this channel was involved in the induction of LTP in different brain areas, such as the hippocampus and amygdala (Wemmie et al., 2002; Du et al., 2014; Buta et al., 2015). In addition, behavioral studies have shown that disrupting or over-expressing ASIC1a in mice might cause alteration in learning and conditioning (Wemmie et al., 2002, 2003, 2004). Recently, we published the researches that demonstrated a role of ASIC1a in metabotropic glutamate (mGlu) receptor-dependent long-term depression (LTD) in the hippocampus (Mango et al., 2017; Mango and Nisticò, 2018). Our results suggest that ASIC1a plays a critical role in intrinsic excitability and mGlu receptor-dependent LTD in CA1 pyramidal neurons of early adult mice. Notably, ASIC1a controls AMPA-GluA1 subunit phosphorylation succeeding mGlu-LTD, suggesting that a functional crosstalk among ASIC1a and AMPA receptors underlies specific forms of synaptic plasticity (Mango et al., 2017).

Here we extend these results by investigating the involvement of ASIC1a in a distinct form of synaptic plasticity. In particular, we studied the NMDA receptor-dependent form of LTD by the use of selective and nonselective ASIC1a blockers, psalmotoxin-1 and amiloride. We have shown that ASIC1a plays a role in NMDA-dependent long-term depression in young and adult hippocampus. Moreover, experiments performed on isolated EPSC_NMDA_ highlight that a functional crosstalk between the two receptors occurs and underlies specific forms of LTD. This interaction was also confirmed by monitoring, during NMDA-LTD experiments, the ASIC1a-mediated currents elicited by puff applications of acidic pH solution. Indeed, ASIC1a currents were increased following chemical LTD induction and were completely abated by PcTx-1 application. It is possible to hypothesize that in the absence of PcTx-1, LFS induces LTD of AMPA-mediated currents; whereas, in the presence of PcTx-1, LFS induces LTP of NMDA- or ASIC1a-mediated currents. Thus, when combining the LTD of AMPA-mediated currents and the potentiation of either NMDA-mediated or ASIC1a-mediated currents that occurs following LFS or NMDA application in the presence of PcTx-1, the end result is attenuation of LTD or unmasking of potentiation.

In any case, here we demonstrate that an interplay between ASICs and glutamate receptors (see also Mango et al., 2017) underlies various forms of synaptic plasticity, even though the precise mechanisms mediating these interactions still remain unclear. Gao et al. (2015) have previously demonstrated interplay between ASIC1a and NMDA receptor function. In particular, they show that ASIC1a activity plays a key role in facilitating the opening of NMDA receptor channel, whereas inhibition of ASIC1a impaired NMDA receptor function at physiological pH (Gao et al., 2015). Importantly, a recent paper has shown an increase of the NMDA receptor current following ASIC1a activation, which is mediated by the NR2 subunit (Ma et al., 2019). Based on these results, here we suggest that ASICs’ activation is involved in NMDA receptor-dependent LTD. It can be hypothesized that under synaptic activity, the opening of ASIC1a might contribute to depolarize the postsynaptic cell thus allowing Ca^2+^ to flow into the NMDA receptor within the dendritic spine and trigger LTD.

Overall, this work further supports a role for ASICs in regulating synaptic function and potentially cognitive processes. Future studies are required to elucidate how the interplay among ASICs and NMDA receptors might contribute to normal and pathological conditions. Interestingly, it is known that drugs that increase synaptic activity, such as NMDA receptor agonists or modulators, have been extensively explored as treatments to ameliorate memory function (for review see Müller et al., 1994; Chazot, 2004). In this context, also ASICs might be considered as potential therapeutic targets in neurodegenerative disorders (Xiong et al., 2008; Radu et al., 2016).

## Ethics Statement

All experiments followed international guidelines on the ethical use of animals from the European Communities Council Directive (2010/64/EU).

## Author Contributions

DM designed the research, performed experiments and wrote the paper. RN designed the research and wrote the paper.

### Conflict of Interest Statement

The authors declare that the research was conducted in the absence of any commercial or financial relationships that could be construed as a potential conflict of interest.

## Abbreviations

aCSF: artificial cerebrospinal fluid
ASIC: acid sensing ion channel
EPSC: excitatory postsynaptic current
LFS: low-frequency stimulation
LTD: long-term depression
PcTx1: psalmotoxin-1.
